# The complete mitogenome of the trilobite beetle, *Platerodrilus* sp. (Elateroidea: Lycidae)

**DOI:** 10.1080/23802359.2016.1219626

**Published:** 2016-09-18

**Authors:** Juan E. Uribe, Jorge Gutiérrez-Rodríguez

**Affiliations:** Museo Nacional De Ciencias Naturales (MNCN-CSIC), Madrid, Spain

**Keywords:** Coleoptera, Elaterimorpha, mitogenome, next-generation sequencing

## Abstract

The nucleotide sequence of the complete mitogenome of the trilobite beetle, *Platerodrilus* sp. was determined. This is the first complete mitogenome reported for the family Lycidae (Elateroidea: Coleoptera). The newly determined sequence is 16,394 bp long and shows a relatively high AT content (76.2%). The gene arrangement of the trilobite beetle mt genome is the same found in other related beetle mitogenomes. The reconstructed tree recovered Elateroidea as a strongly supported monophyletic group but could not resolve phylogenetic relationships among studied elateroid families, including Lycidae.

## Introduction

The genus *Platerodrilus* Pic, 1921 belongs to the family Lycidae Laporte, 1836, within the superfamily Elateroidea Leach, 1815. This genus is distributed along the Southeast Asia, showing its higher species diversity in Indo-Burma, Sundaland, and Philippines (Masek & Bocak 2014; Masek et al. 2015). In this genus, females do not complete metamorphosis, remaining in a larviform stage, whereas males complete their normal development (Bocak & Matsuda 2003). Interestingly, neoteny appears independently in several families within Elateoroidea (Bocakova et al. 2007). The superfamily comprises 17 recognized families (Bouchard et al. 2011), of which only five have complete mitogenomes available at GenBank.

Here, the first complete mitogenome for a representative of the family Lycidae was sequenced following the methods described in Uribe et al. (2016). The mitogenome was amplified and sequenced using specific primers: PLcox3F (5′AAGCTATTTTCACAGAGGATTATCACC-3′); PLcox3R (5′TTACGTGTATTCCATGAAATCCTGT TGC-3′); PLrrnLF (5′-TTTAAAAGACGAGAAGACCCTATAGAG-3′); and PLrrnLR (5′-ATTTTAATCCAACATCGAGGTCGCAAAC-3′). These long-PCR products were purified by ethanol precipitation, pooled together in equimolar concentrations, and subjected to massive parallel sequencing. An indexed library was constructed using the NEXTERA XT DNA library prep Kit (Illumina, San Diego, CA) at AllGenetics (A Coruña, Spain). The indexed libraries were run in a single lane in an Illumina-MiSeq2000 (150 Pair-ended) at Macrogen (Seoul, Korea).

The assembling of the complete mitogenome of *Platerodrilus* sp. (voucher MNCN/DNA: 86739; Locality: Indonesia, Kalimantan, Tumbang Samba (−1.180°, 112.432°)) was performed in the TRUFA webserver (Kornobis et al. 2015). The final assembly was used as reference genome to map the original (raw) reads with a minimum identity of 100% using Geneious^®^ 8.0.3. A total of 27,703 reads mapped onto the reference genome, which was 16,394 pb long and had a mean coverage of 255×. As most animals (Gissi et al. 2008), the mitogenome of *Platerodrilus* sp. encodes for 37 genes. The gene order of the mitogenome is similar to other closely related beetles (Arnoldi et al. 2007; Sheffield et al. 2008). The overall A + T composition of the mitogenome was relatively high 76.2%, as well as other species included in Elateroidea. Six protein-coding genes had incomplete stop codons that require polyadenylation (such as *cox1*, *cox3*, *nad3*, *nad5*, *nad4*, and *cob*).

**Figure 1.**
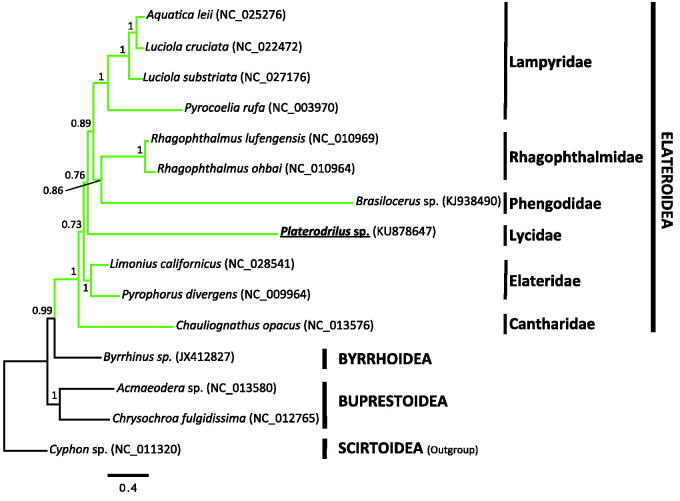
Phylogenetic relationships of Elateroidea based on 13 protein-coding genes at the amino acids level of complete or nearly complete mitogenomes (2954 positions long). The NCBI accession numbers of each mt genome used are shown in parenthesis.

We performed a Bayesian inference as implemented in PhyloBayes MPI 1.5a (Lartillot et al. 2013) using the site-heterogeneous mixture CAT-GTR model (Lartillot & Philippe 2004). The newly determined mitogenome sequence was aligned in Translator X (Abascal et al. 2010) with orthologous sequences corresponding to complete or nearly complete mitogenomes available in GenBank for Elateroidea and closely related superfamilies with Scirtoidea as outgroup. The reconstructed phylogeny showed Byrrhoidea as closest sister group of Elateroidea with robust statistical support (Figure 1). Elateroidea is recovered as a monophyletic group with high support. Within this superfamily, those families with more than one representative were recovered as monophyletic with strong statistical support, but phylogenetic relationships among families were largely unresolved, as in recent studies based on similar sequence data (Timmermans et al. 2016). Interestingly, Lycidae and Phengodidae showed long branches, which may be affecting resolution.
